# From genomes to genotypes: molecular epidemiological analysis of *Chlamydia gallinacea* reveals a high level of genetic diversity for this newly emerging chlamydial pathogen

**DOI:** 10.1186/s12864-017-4343-9

**Published:** 2017-12-06

**Authors:** Weina Guo, Martina Jelocnik, Jing Li, Konrad Sachse, Adam Polkinghorne, Yvonne Pannekoek, Bernhard Kaltenboeck, Jiansen Gong, Jinfeng You, Chengming Wang

**Affiliations:** 1 Jiangsu Co-Innovation Center for Prevention and Control of Important Animal Infectious Diseases and Zoonoses, Yangzhou University College of Veterinary Medicine, Yangzhou, Jiangsu 225009 People’s Republic of China; 2 College of Animal Science, Anhui Science and Technology University, Maanshan, Anhui China; 3 Centre for Animal Health Innovation, Faculty of Science, Health, Education and Engineering, University of the Sunshine Coast, Maroochydore, QLD Australia; 4 Institute of Bioinformatics, Friedrich-Schiller-Universität Jena, Jena, Germany; 5 Department of Microbiology, University of Amsterdam, Amsterdam, The Netherlands; 6 College of Veterinary Medicine, Auburn University, Auburn, AL USA; 7 Poultry Institute, Chinese Academy of Agricultural Sciences, Jiangsu Co-Innovation Center for Prevention and Control of Important Animal Infectious Diseases and Zoonoses, Yangzhou, Jiangsu China

**Keywords:** *Chlamydia gallinacea*, Whole-genome sequence, Comparative genomics analysis, MLST, Phylogenetic analysis

## Abstract

**Background:**

*Chlamydia* (*C.*) *gallinacea* is a recently identified bacterium that mainly infects domestic chickens. Demonstration of *C. gallinacea* in human atypical pneumonia suggests its zoonotic potential. Its prevalence in chickens exceeds that of *C. psittaci*, but genetic and genomic research on *C. gallinacea* is still at the beginning. In this study, we conducted whole-genome sequencing of *C. gallinacea* strain JX-1 isolated from an asymptomatic chicken, and comparative genomic analysis between *C. gallinacea* strains and related chlamydial species.

**Results:**

The genome of *C. gallinacea* JX-1 was sequenced by single-molecule, real-time technology and is comprised of a 1,059,522-bp circular chromosome with an overall G + C content of 37.93% and sequence similarity of 99.4% to type strain 08-1274/3. In addition, a plasmid designated pJX-1, almost identical to p1274 of the type strain, except for two point mutations, was only found in field strains from chicken, but not in other hosts. In contrast to chlamydial species with notably variable polymorphic membrane protein (*pmp*) genes and plasticity zone (PZ), these regions were conserved in both *C. gallinacea* strains. There were 15 predicted *pmp* genes, but only B, A, E1, H, G1 and G2 were apparently intact in both strains. In comparison to chlamydial species where the PZ may be up to 50 kbp, *C. gallinacea* strains displayed gene content reduction in the PZ (14 kbp), with strain JX-1 having a premature STOP codon in the *cytotoxin* (*tox*) gene, while *tox* gene is intact in the type strain. In multilocus sequence typing (MLST), 15 *C. gallinacea* STs were identified among 25 strains based on cognate MLST allelic profiles of the concatenated sequences. The type strain and all Chinese strains belong to two distinct phylogenetic clades. Clade of the Chinese strains separated into 14 genetically distinct lineages, thus revealing considerable genetic diversity of *C. gallinacea* strains in China.

**Conclusions:**

In this first detailed comparative genomic analysis of *C. gallinacea*, we have provided evidence for substantial genetic diversity among *C. gallinacea* strains. How these genetic polymorphisms affect *C. gallinacea* biology and pathogenicity should be addressed in future studies that focus on phylogenetics and host adaption of this enigmatic bacterial agent.

**Electronic supplementary material:**

The online version of this article (10.1186/s12864-017-4343-9) contains supplementary material, which is available to authorized users.

## Background

The obligate intracellular bacteria in the genus *Chlamydia* are globally widespread and represent successful pathogens that infect a wide range of animals as well as humans. However, some of them are frequently overlooked as these infections typically remain latent and only rarely lead to overt clinical signs. For a long time, *Chlamydia* (*C.*) *psittaci*, an avian pathogen with well-documented zoonotic potential, was considered the only chlamydial species infecting domestic and wild birds. However, recent reports showed that *C. gallinacea* and *C. avium* are two emerging chlamydial agents that can also be involved in avian chlamydiosis [1]. To date, *C. avium* has been detected in pigeons and psittacine birds, while *C. gallinacea* has been mainly detected in chickens, ducks, guinea fowl, turkey, backyard poultry and cattle [2, 3]. Interestingly, the high prevalence of *C. gallinacea* in poultry flocks across Europe and China determined by PCRs surpassed that of *C. psittaci* [4, 5]. This organism is known to occasionally be in transmission with *C. psittaci* in the same flock and also can co-infect individuals [6, 7]. Beyond the potential role of this emerging pathogen in animal health, an earlier study of an outbreak of atypical pneumonia in a slaughterhouse, where workers were exposed to *C. gallinacea*-infected chickens, raised questions over its zoonotic potential as well [8].

Whole-genome sequencing and subsequent comparative genomic analysis has become standard in analysis of the biology, virulence factors, evolution and phylogenetic relationships of chlamydial organisms [9, 10]. While there is plentiful genomic data on the related chlamydial species, the only completely assembled genomic sequence of *C. gallinacea* currently available is that of the type strain 08-1274/3, which was isolated from a chicken in France [11]. So far, this limited genomic information for *C. gallinacea* has allowed only little insight into its developmental cycle and potential virulence factors. Likewise, intra-species genetic diversity and phylogenetic relationships have yet to be investigated. Little information available from partial multi-locus sequence analysis (MLSA) of five *C. gallinacea* strains revealed limited genetic diversity within the species [12]. However, this contrasts with the findings of our own genotyping studies targeting the *ompA* gene, which encodes the chlamydial major outer membrane protein (MOMP), where we found 13 diverse *ompA* genotypes of *C. gallinacea* in Chinese poultry [4].

In the present study, we describe the second whole-genome sequence (WGS) of *C. gallinacea*, which originates from the Chinese chicken isolate JX-1, and report findings from comparative genomic analysis between *C. gallinacea* strains and closely related species in the genus *Chlamydia*. To understand the epidemiology and genetic diversity of *C. gallinacea* infections in chickens, we conducted previously described *Chlamydiales* multi-locus sequence typing (MLST) [13] on 23 *C. gallinacea*-positive samples from nine farms located in nine provinces across China. This enabled us to provide a detailed description of genomic features and assess naturally occurring genetic diversity of this pathogen.

## Methods

### Description of *C. gallinacea* isolate JX-1 and clinical samples used in this study

*C. gallinacea* JX-1 strain, used for genome sequencing and plasmid characterization, was isolated from a cloacal swab of an asymptomatic chicken in the Jiangxi province of China [4]. In the present study, we also used DNA from 45 previously tested *C. gallinacea*-positive clinical swabs taken from oral and cloacal anatomical sites of chickens, pigeons, ducks and geese from various farms across China [4] (Additional file 1: Table S1). Ethics approval was not needed as the DNA used in this study was extracted from the chickens in a previous study [4].

### Whole-genome sequencing and assembly

*C. gallinacea* strain JX-1 was propagated via yolk sac inoculation on a 7-day-old chicken embryo followed by yolk membrane harvesting, in order to perform genomic DNA extraction using the QIAgen® DNA Mini Kit (Qiagen, Valencia, CA, USA). The obtained total DNA was subjected to quality control, by running 1 μl of DNA on an agarose gel and quantification by Qubit. The genome of *C. gallinacea* JX-1 was sequenced by Single-Molecule, Real-Time (SMRT) technology at the Beijing Novogene Bioinformatics Technology Co., Ltd. (China). SMRT Analysis 2.3.0 was used to filter low-quality reads, following assembly into a single gap-free contig using filtered reads. Low-quality reads were filtered by the SMRT Analysis v2.3.0 software, and then the genome was subjected to de novo assembly by the SMRT portal software according to the valid sequencing data. The draft *C. gallinacea* JX-1 genome was automatically annotated using the NCBI Prokaryotic Genomes Annotation Pipeline (NCBI_PGAP), and the genome sequence was deposited in the NCBI database under GenBank accession number CP019792.

### *C. gallinacea* plasmid screening

In order to assess whether the *C. gallinacea* JX-1 carries a plasmid, 16 paired primers were designed based on the plasmid sequence of type strain 08-1274/3 (Additional file 2: Table S2) to amplify the complete plasmid sequence. PCR conditions and reaction mixes are described in the section below. Each amplified fragment was purified using the QIAquick PCR Purification Kit (Qiagen), and sent for Sanger sequencing to GenScript, Jiangsu, Nanjing, China. The chromatograms of the sequenced plasmid fragments were mapped against the p1274 sequence, and the complete *C. gallinacea* JX-1 plasmid (pJX-1) was extracted and annotated using RAST [14] and deposited in GenBank under accession number CP019793. We have also screened the *C. gallinacea*-positive clinical samples (Additional file 1: Table S1) for plasmid presence by amplifying a 661 bp fragment of the plasmid’s CDS1 (integrase) using primer pair plaF1 and plaR1 of the plasmid (Additional file 2: Table S2).

### Macroscopic comparative genomic and phylogenetic analyses

The genome of *C. gallinacea* JX-1was compared in-depth to the reference genome of type strain 08-1274/3, as well as to publicly available genomes of other related chlamydial species, i.e. *C. avium* 10 DC88 (NZ_CP006571.1), *C. pecorum* E58 (CP002608), *C. psittaci* 6 BC (CP002586.1) and *C. abortus* S26/3 (CR848038.1). Pairwise genomic comparison was performed using the Artemis Comparison Tool (ACT) [15], and Geneious 9 [16] using alignments produced with progressive Mauve [17] and MAFFT [18]. The genomic regions of interest and/or loci were extracted from the analyzed genomes and aligned, in order to be used for further nucleotide and/or translated protein sequence analyses performed using DNASp 5.0 [19], as well as BLAST (https://blast.ncbi.nlm.nih.gov/Blast.cgi). In addition, we have also used open source TMHMM Server v. 2.0 (available from http://www.cbs.dtu.dk/services/TMHMM/) which predicts transmembrane helices in proteins, to predict chlamydial inclusion membrane proteins with a presence of bilobed hydrophobic domains using translated *C. gallinacea* hypothetical gene sequences. A mid-point rooted phylogenetic tree constructed from the alignment of all identified *C. gallinacea pmp* genes from both strains used in this study was generated with PhyML with 1000 bootstrap repetitions [20], as implemented in Geneious 9. Figures of the whole-genome comparison and specific genomic regions using blastn and tblastx algorithms were generated with Brig [21] and EasyFig [22], while the graphical representation of the *C. gallinacea* JX-1 genome and its elements was generated with the DnaPlotter [23].

The phylogenetic relationship of the two *C. gallinacea* strains was examined through comparison to each other and related chlamydial species using an 11.2 kbp alignment of concatenated sequences. The concatenated sequence consisted of 12 partial and full-length conserved chlamydial phylogenetic markers that were concatenated in the following order: six MLST house-keeping gene fragments (*gat*A, *hfl*X, *gid*A, *eno*A, *hem*N, *fum*C), and full-length major outer membrane protein gene *omp*A, DNA-directed RNA polymerase subunit beta gene *rpo*B, 50S ribosomal protein L3 gene *rpl*C, 50S ribosomal protein L4 gene *rpl*D, DNA recombination/repair protein gene *rec*A, and tyrosine—tRNA ligase gene *tyr*S. In addition to the two *C. gallinacea* genomes used in this study, each of these sequences were extracted from the genomes of the following related species: *C. avium* 10 DC88 (NZ_CP006571.1), *C. caviae* GPIC (NC_003361.3), *C. felis* F/C-56 (NC_007899.1), *Candidatus C. ibidis* 10-1398/6 (NZ_APJW00000000.1), *C. pneumoniae* LpColN (NC_017285.1), *C. pecorum* E58 (CP002608), *C. psittaci* 6 BC (CP002586.1), *C. trachomatis* AHAR-13 (CP000051.1), *C. muridarum* Nigg (NC_002620.2), *C. suis* MD56 (NZ_KI538658.1) and *C. abortus* S26/3 (CR848038.1). A mid-point rooted maximum-likelihood phylogenetic tree was constructed using PhyML with 1000 bootstrap repetitions, as integrated in Geneious 9.

### MLST of *C. gallinacea*

In this study, we performed a complete MLST, based on a previously published scheme for chlamydiae [13]. The primers used to amplify seven *C. gallinacea*-specific house-keeping (HK) genes were designed in this study based on the sequence of *C. gallinacea* type strain 08-1274/3 and other related chlamydial species (Additional file 3: Table S3).

PCR amplification of the seven HK genes (as well as plasmid fragments) was performed in a LightCycler 480-II real-time PCR platform using a high-stringency 18-cycle step-down temperature protocol (Additional file 4: Table S4) as described [3, 4]. *C. gallinacea* JX-1 DNA was used as a positive control, while ultrapure H_2_O was used as a negative control in each assay. The PCR products were electrophoresed through 2% agarose gel and purified using the QIAquick PCR Purification Kit (Qiagen) for automated DNA sequencing (GenScript, Jiangsu, Nanjing, China).

After optimization and development, the *C. gallinacea* MLST was applied to sequences of a total of 45 *C. gallinacea*-positive oral and cloacal swabs from chickens (*n* = 20), hens (*n* = 4), ducks (*n* = 12), pigeons (*n* = 6), and geese (*n* = 3), collected in different provinces of China (Additional file 1: Table S1).

*C. gallinacea* MLST and phylogenetic analysis were performed using DNASp 5.0 and Geneious 9. Briefly, forward and reverse chromatograms for each sequenced HK gene fragment were aligned and trimmed, and the fragment sequence for that allele was obtained. Allele and sequence type (ST) assignment for 25 *C. gallinacea* strains were determined and deposited at http://pubmlst.org/chlamydiales/ [24] (Additional file 5: Table S5).

Sequences of individual genes and concatenated gene sets were aligned using ClustalX. DnaSP 5.0 was used to analyze sequence polymorphisms by determining the number of synonymous (d_s_) and non-synonymous (d_n_) substitutions per site, Jukes-Cantor corrected, the number of polymorphic sites and haplotypes (Additional file 5: Table S5).

Best-fit models of nucleotide substitution for our data set were estimated by considering eleven substitution (nst = 11) models using jModelTest v.2.2. [25]. A Bayesian phylogenetic tree using concatenated MLST sequences of 25 *C. gallinacea* strains was constructed with MrBayes [26] with the HKY + I model, as implemented in Geneious 9. Run parameters included four Markov Chain Monte Carlo (MCMC) chains with a million generations, sampled every 1000 generations and with the first 10,000 trees were discarded as burn-in. The *C. avium* MLST sequence was used as an outgroup.

## Results

### Description of the *C. gallinacea* JX-1 genome

**Fig. 1:**
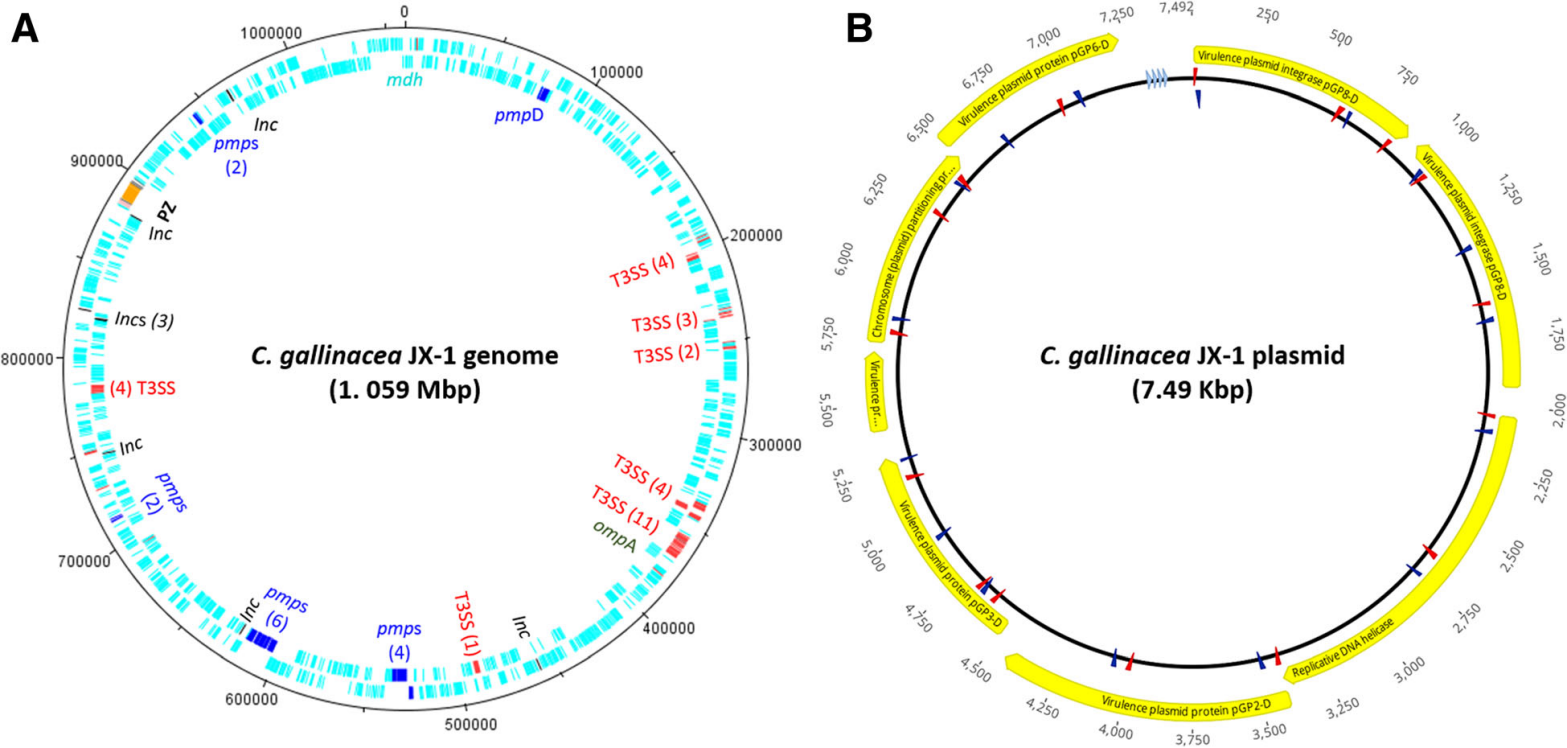
Circular representation of the *C. gallinacea* JX-1 genome and its cognate plasmid pJX-1. **a** JX-1 genome: First ring denotes CDSs (in light blue) in forward direction, while the second ring denotes CDSs in reverse direction. Genomic location of T3SS, *pmp*, *Inc.*, and *omp*A genes, as well as the PZ are also outlined on the JX-1 genome plot. The genome start is denoted by the malate dehydrogenase gene (*mdh*) at 0 position. Image was generated with the DNAplotter. **b** Graphical representation of the p*Cgall* JX-1 and its CDSs, including plasmid primer locations. The light blue arrows denote the 22 bp tandem repeat units

Using SMRT sequencing technology, we have completely sequenced and assembled the genome of *C. gallinacea* strain JX-1. The whole genome is sized 1,059,522 bp with an overall GC content of 37.93%, encompassing 957 predicted CDSs that account for 91.21% of the genome and a 7.49 kbp plasmid (Table 1, Fig. 1). Alignment to the reference genome of *C. gallinacea* type strain 08-1274/3 confirmed 100% chromosome coverage for our newly described JX-1 genome.

**Table 1:** Description of *Chlamydia gallinacea* JX-1 genome

Strain	*Chlamydia gallinacea* JX-1
Clinical manifestation and anatomical site sample type	Asymptomatic/Cloacal swab
Host and country of origin	Chicken, China
Total No. of filtered reads	66,564
Average read length	11,688 bp
Average read depth	100 ×
Genome size (bp) and % GC	1,059,522 bp / 37.93% GC
No. of predicted CDSs	957
Chlamydial plasmid and size	Present: pJX-1 (7.49 Kbp)
nc RNAs	39 tRNAs, two 5S rRNA, and single copy of 16S rRNA and 23S rRNA
Pyrimidine genes	Present (*pyr*G, *pyr*E, *pyr*H, *ndk*)
Biotin operon	Present (*bio*ADFB, bioY)
Tryptophan operon	Absent
% DNA sequence identity *C. gallinacea* 08-1274/3	99.4%
Accession number	CP019792

**Fig. 2:**
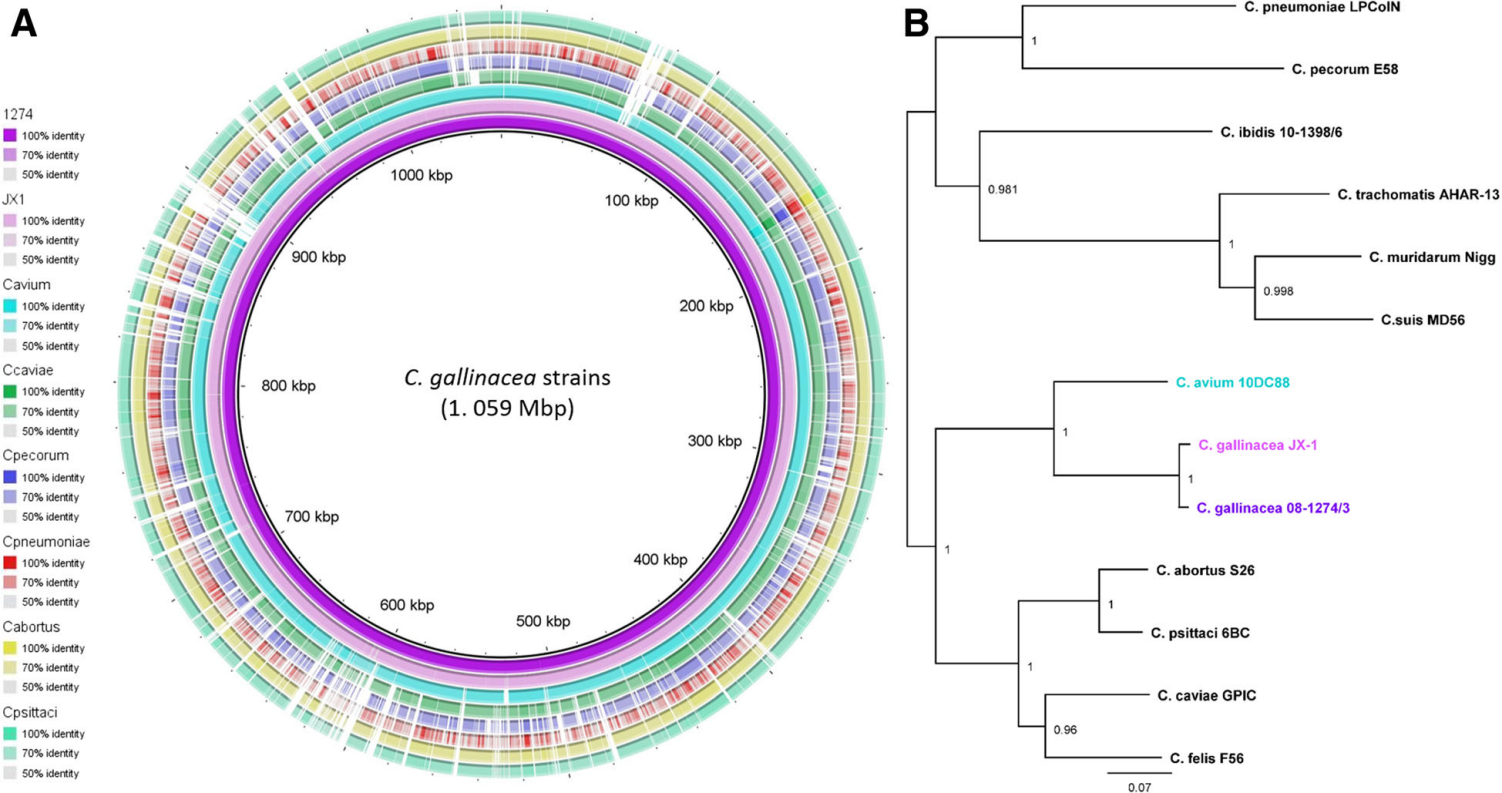
*C. gallinacea* genome comparisons and phylogenetic analyses. **a** Whole genome BLAST comparisons between the *C. gallinacea* and the related species based on translated nucleotide identity (tblastx algorithm). **b** The mid-point rooted maximum-likelihood phylogenetic analyses of the 12 conserved phylogenetic markers, resulting in 11.2 Kbp concatenated sequence alignment of the 13 chlamydial species. Bootstrap values are displayed on the tree nodes

With the availability of the JX-1 genome, we were able to evaluate genome similarities and differences between *C. gallinacea* strains, but also to related chlamydial species (Fig. 2a). The two *C. gallinacea* genomes are virtually identical to each other (99.4% identity) and share the highest similarity with the *C. avium* genome (~78.9% sequence similarity) based on whole-genome MAFFT alignment. Comparison to the genomes of the other chlamydial species revealed more pronounced differences, mainly in the PZ and Pmp clusters, however with a remarkable overall chlamydial genome synteny (Fig. 2a). Phylogenetically, although closely related, the two *C. gallinacea* strains, grouped in a larger clade with their closest relative, *C. avium* (Fig. 2b).

The *C. gallinacea* genomes of field strain JX-1 and type strain 08-1274/3 exhibited high sequence similarity (99.4% identity) with ~6250 SNP differences between the two strains, while maintaining synteny and gene order. Both *C. gallinacea* genomes also contained the hallmark genomic features of chlamydiae, such as the highly conserved Type III Secretion System (T3SS), clusters of *pmp* genes, inclusion protein genes (*incs*), and a PZ (Fig. 1a) [27].

With SNPs evenly distributed along the chromosome, the major genetic differences between strains JX-1 and 08-1274/3 are presented in Table 2. In comparative genomic analysis, we have identified genes with high density of SNPs (with at least 5% total length sequence dissimilarity between the two strains), Interestingly, besides the previously recognized highly variable *omp*A gene, most of the remaining genes with high numbers of SNPs were annotated as metabolic genes (Table 2). Highly variable CDS GM000264, annotated as a hypothetical protein, appears to harbor a *C. gallinacea*-specific sequence based on BLAST searches, with only 20% similarity to a conserved hypothetical protein of *C. psittaci*. The putative product, however, does not seem to have a conserved domain. None of these identified genes appears to be under positive selection with the majority of accumulating SNPs being synonymous (Table 2). However, due to only two strains analyzed, at present we cannot accurately assess the selection on these genes.

**Table 2:** Sequence analysis of *C. gallinacea* polymorphic genes

Locus tag and length (bp) in *C. gallinacea* JX-1	Predicted product	% DNA sequence similarity	Total No. of polymorphisms	No. of non-synonymous substitutions	No. of synonymous substitutions
GM000925 (1581 bp)	Lysine-tRNA ligase	95.1%	77 SNPs	11	65
GM000895 (873 bp)	Serine/threonine protein phosphatase	93.2%	59 SNPs	12	53
GM000890 (2631 bp)	Alanyl-tRNA synthetase	92.5%	198 SNPs	36	162
GM000889 (3252 bp)	Transcription-repair coupling factor	94.2%	190 SNPs	37	153
GM000888 (924 bp)	Uroporphyrinogen decarboxylase (hemE)	92.2%	72 SNPs	9	63
GM000887 (1374 bp)	Coproporphyrinogen oxidase (hemN)	93.0%	96 SNPs	31	65
GM000705 (1206 bp)	Major outer membrane protein, porin	86.7%	161 (104 SNPs and 57 indel)	38	60
GM000539 (1356 bp)	Sodium/alanine symporter family protein	95.2%	65 SNPs	12	53
GM000349 (1236 bp)	Cell wall hydrolase	91.7%	102 (87 SNPs and 15 indel)	41	45
GM000348 (1179 bp)	Phage T7 tail fiber family protein	80.3%	232 (133 SNPs and 99 indel)	59	74
GM000289 (2409 bp)	Glycogen phosphorylase	94.1%	142 SNPs	20	122
GM000288 (1290 bp)	Dihydrolipoamide acetyltransferase component	91.1%	115 SNPs	40	75
GM000264^a^ (1765 bp)	Hypothetical protein	88.3%	202 (175 SNPs and 27 indel)	67	108

^a^C. gallinacea-specific sequence based on BLAST search

The *C. gallinacea* strains possess a chlamydial T3SS comprised of a total of 36 genes encoding T3SS structural components, chaperones and secreted effectors (Additional file 6: Table S6). T3SS genes of the two *C. gallinacea* strains were highly conserved with 98–100% sequence similarity, in stark contrast to the previously described genetic diversity in these genes in related chlamydial species such as *C. psittaci* [28], *C. pecorum* [29] and human *C. trachomatis* [30].

We have also assessed the predicted Inclusion membrane proteins (Incs) for *C. gallinacea*, as during early infection the inclusion membrane modified by the insertion of a number of type III secreted effector proteins, and the inclusion proteins play a significant role [31]. Using open source prediction software TMHMM Server v. 2.0 using a cut-off of more than 40 amino acids in the bi-lobed hydrophobic domain, we have predicted a total of 29 putative Inc’s with two transmembrane domains, besides the two annotated IncA, and IncB and IncC; five with four transmembrane domains; and three with six transmembrane domains (Additional file 7: Table S7; Additional file 8: Figure S1). This number of predicted Incs is comparable to that observed in the related chlamydial species [31].

### Variation in the *pmp* genes: gene truncations rather than SNP accumulation

**Fig. 3:**
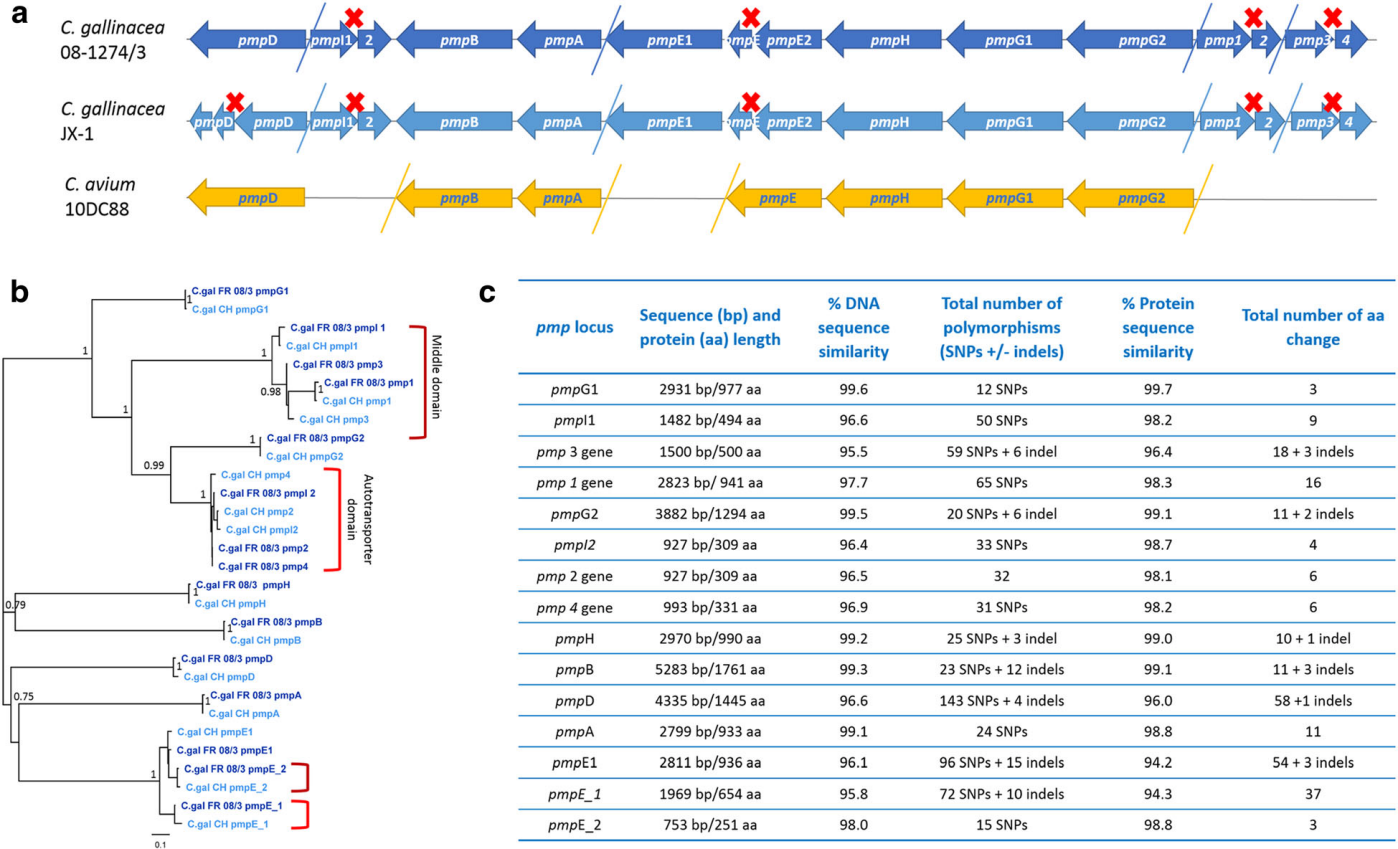
Sequence and phylogenetic analyses of *C. gallinacea pmps*. **a** Schematic representation of *pmp* gene order in the *C. gallinacea* and *C. avium* genomes. Red X denotes STOP codon. **b** A mid-point rooted maximum likelihood phylogenetic tree of the *C. gallinacea pmp* genes. Autotransporter and middle *pmp* gene domains are denoted with brackets. **c** Sequence analyses of the *C. gallinacea pmp* genes and Pmp protein

In the *C. gallinacea* genomes, the *pmp* genes were found to form two major clusters. In our analysis, we predicted a total of 15 *pmp* genes, however only B, A, E1, H, G1 and G2 appear to be intact in both *C. gallinacea* strains (Fig. 3). The *pmp*D gene of strain JX-1 was found to have a premature STOP codon (Fig. 3a). The remaining four *pmp* genes also had a premature STOP codon in both strains, which was predicted to truncate the encoded proteins before their respective C-terminal autotransporter domains as based on BLAST and CDD (Conserved domains) BLAST analysis. BLAST and phylogenetic analyses confirmed that the majority of the predicted *pmp* genes in the *C. gallinacea* genomes are paralogs of *pmp*G gene lineage (Fig. 3b).

In terms of the number of intact *pmp* genes, *C. gallinacea* was closest to *C. avium* (*n* = 7), while the related chlamydial species harbor between 14 (*C. abortus*) and 22 *pmp* genes (*C. psittaci*). In contrast to the related chlamydial species where these genes are major contributors of SNPs [29, 32], *pmp* genes of the two analysed *C. gallinacea* strains were conserved, with overall sequence similarity ranging from 95.5 to 99.6%. Comparable levels of similarity were observed when comparing the pmp amino acid sequences (Fig. 3c).

### The *C. gallinacea* plasticity zone (PZ)

**Fig. 4:**
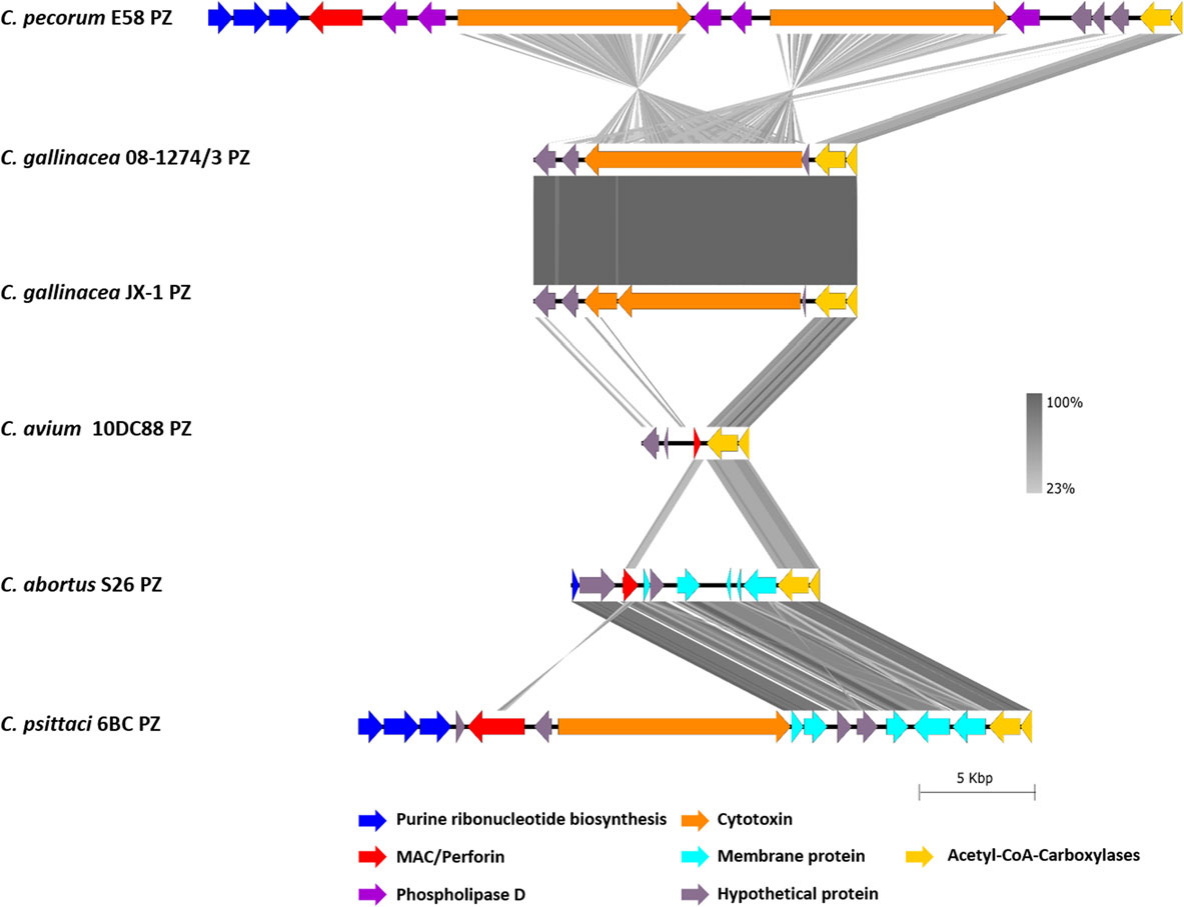
Graphical representation of the gene content in chlamydial PZs, including the two *C. gallinacea* strains analyzed in this study. Colored arrows in the legend denote PZ genes according to their function, while the grey shading scale denotes % sequence similarity. Image was generated with Easyfig using tblastx comparison

The PZ, notoriously known for harboring key virulence genes of chlamydiae, has been considered as the region of most extensive genetic differences between chlamydial genomes [27]. In comparison to the related chlamydial species where the PZ may be up to 50 kbp in size (e.g. in *C. muridarum* and *C. trachomatis*), *C. gallinacea* displayed gene content reduction in this region (14 kbp), similar to *C. abortus* (12 kbp). In our analysis, *C. avium* appeared to have the most reduced PZ (4.6 kbp) (Fig. 4). The PZ of two *C. gallinacea* strains included three hypothetical proteins, two acetyl-co-carboxylases and a single copy of the chlamydial *cytotoxin* (*tox*) gene, but remained highly conserved with 99.2% sequence similarity. Interestingly, *C. gallinacea* JX-1 had a premature STOP codon in the cytotoxin (*tox*) gene, while *tox* appeared to be intact in the type strain 08-1274/3 (Fig. 4).

### Molecular characterization of plasmid pJX-1 and its distribution in *C. gallinacea* strains

Newly characterised plasmid pJX-1 of *C. gallinacea* was 7.49 kbp in length, sharing an identical annotation with eight CDSs and four 22 bp tandem repeats with plasmid p1274 of the type strain 08-1274/3 (Fig. 1b). Briefly, in pJX-1, CDSs 1 (pGP8), 2 (pGP8), 3 (pGP1), and 7 (*par*A) were denoted as putative integrase, helicase, and a partitioning plasmid proteins, respectively, while the CDSs 4 (pGP2), 5 (pGP3), 6 (pGP4), and 8 (pGP6) were denoted as putative chlamydia-specific plasmid virulence proteins, as previously described for related chlamydial plasmids [33, 34]. The sequence of pJX-1 was 99.9% identical to p1274, except for two point mutations at positions 6573 (C changed to T) and 7170 (C changed to A) in CDS 8 (pGP6). Further PCR examination of *C. gallinacea*-positive clinical samples revealed that pJX-1 was detected in all the 24 oral/cloacal swab samples from chickens, while clinical samples from other avian hosts remained negative (data not shown).

### MLST of *C. gallinacea* reveals genetic diversity among strains in chickens

In order to obtain a snapshot of the genetic diversity of this emerging pathogen, we have applied our newly developed *C. gallinacea*-specific MLST to a range of *C. gallinacea*-positive clinical samples collected from birds (Additional file 1: Table S1). Unfortunately, due to insufficient amounts of *C. gallinacea* DNA, MLST was only successful in 23 cloacal and oral clinical samples from 3 hens and 20 chickens.

Using a set of 25 sequences, including the MLST sequences obtained from the two sequenced *C. gallinacea* genomes, sequence analysis of individual as well as concatenated gene fragments confirmed the evolutionary conservation of the HK genes, as all alleles were under purifying selection with dn/ds ratios < 1(Table 3). The highest number of mutations was noted in *hem*N (41 SNPs) and *eno*A (25 SNPs), resulting mainly in synonymous substitution, whereas *gat*A had none. *eno*A and *opp*A3 were the most diverse loci as they both occurred in six allelic variants (Table 3). The concatenated sequences (further used for phylogenetic analysis) and its cognate MLST allelic profiles resulted in a total of 15 haplotypes or *C. gallinacea* STs (Table 3).

**Table 3:** Sequence analysis of *C. gallinacea* MLST alleles for samples denoted in bold in Additional file 1: Table S1

Allele	Total number of mutations (Δnt)	No. of non-synonymous substitutions	No. of synonymous substitutions	N alleles
*gat*A	0	0	0	1
*opp*A_3	5	2	3	6
*hfl*X	3	0	3	4
*gid*A	2	1	1	2
*eno*A	25	1	24	6
*hem*N	41	13	28	3
*fum*C	7	1	6	5
Concatenated (3098 bp)	83	18	65	15

Δnt: No. of polymorphic sites; ^*^d_s_ and d_n_: the average number of synonymous substitutions per synonymous site and non- synonymous substitutions per non-synonymous site, respectively (Jukes – Cantor corrected); N Alleles: No. of unique sequences according to the gene

**Fig. 5:**
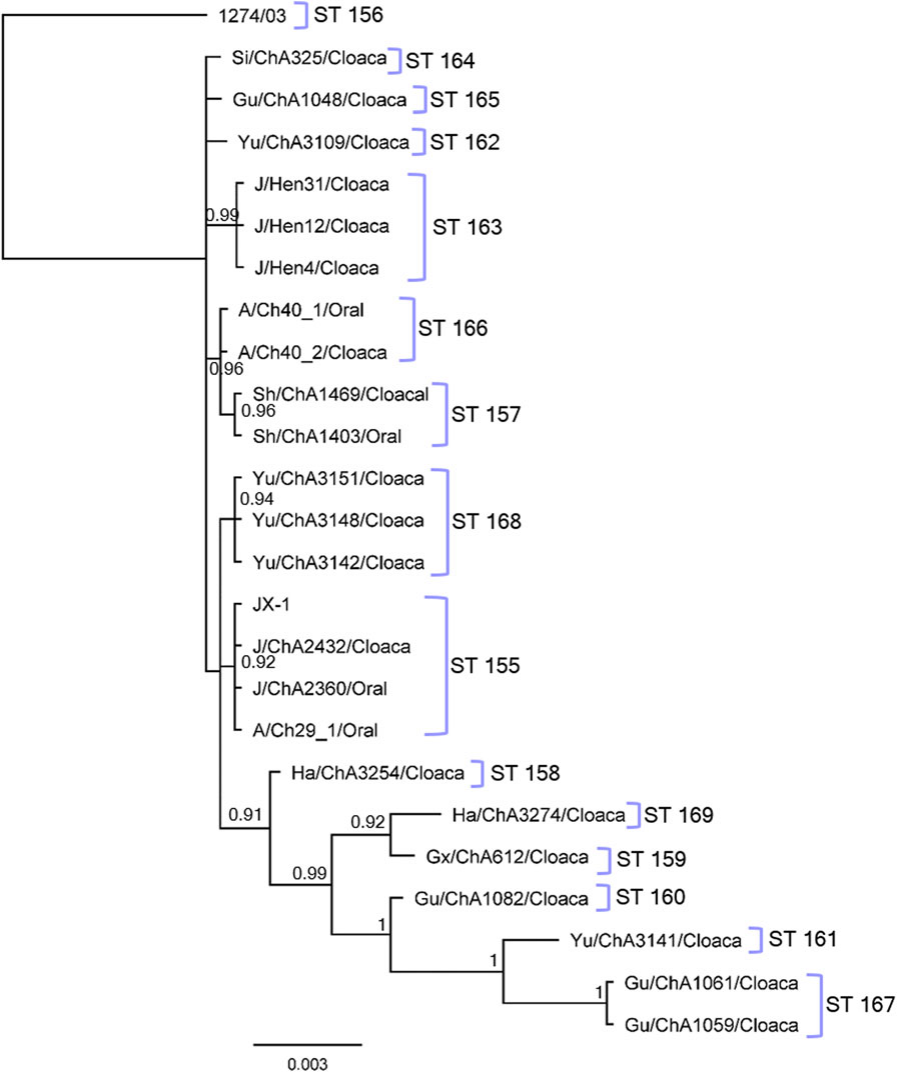
Phylogenetic and cluster analyses of *C. gallinacea* STs. **A**: Bayesian phylogenetic analysis of the concatenated sequences of seven MLST fragments of 25 *C. gallinacea* genotypes. Posterior probabilities > 0.75 are displayed on tree nodes, and STs in a clade are indicated by brackets

To examine the genetic relationships between the *C. gallinacea* strains typed using our MLST method, a mid-point rooted Bayesian phylogenetic tree was constructed using the concatenated HK gene sequences amplified from the 25 strains included. Using the concatenated MLST sequence of the closest relative *C. avium* as an out-group, the phylogenetic tree separated the *C. gallinacea* type strain 08-1274/3 from all Chinese *C. gallinacea* strains into two distinct clades (Fig. 5a). Although in the same well-supported larger clade, *C. gallinacea* strains from China could be further sub-divided into 14 genetically distinct lineages. The phylogenetic tree constructed from the present *C. gallinacea* STs also revealed that: a) The same strain can infect two different anatomical sites in a single host (e.g. A/Ch_40: oral and cloaca); b) the same strain can be found in different animals from the same area (e.g. J/Hen 31, 12 and 4 strains) or geographically distinct areas (e.g. JX-1, J/ChA2432, J/ChA2360 and A/Ch29-1 strains); and c) closely related strains can also be found in geographically distinct areas (e.g. Ha/ChA3274 and Gx/ChA612) (Fig. 5).

## Discussion

In this study, we present the first detailed analysis of *C. gallinacea* genomes. The two *C. gallinacea* genomes studied are compact, syntenic and highly conserved between them, while sharing some of the classical genomic features of *Chlamydia* spp., such as the highly conserved T3SS, *pmp* gene clusters, and a potentially virulence-associated plasmid. The two strains analyzed in this study, JX-1 from China and type strain 08-1274/3 from France form separate phylogenetic lineages within a clade with its closest relative *C. avium*. Using information derived from these genomes and previously described *Chlamydiales* MLST scheme [13], we also adapted a complete *C. gallinacea* MLST scheme and applied it to clinical strains from chickens of various Chinese provinces. The latter analysis revealed that this organism is genetically diverse, indicating the potential for a complex epidemiology similar to other chlamydial species found in animals.

The previously described typical synteny and gene order for chlamydial organisms [9] was also observed in the *C. gallinacea* genomes. The two strains differed in approximately 6250 SNPs, mostly synonymous, which were distributed evenly along the chromosome. The extent of genetic variation in the *omp*A locus was not surprising, as a previous study on *C. gallinacea omp*A typing identified at least 13 genotypes [4]. The *omp*A variation at the variable domains in this species is also consistent with *omp*A diversity seen in other chlamydial pathogens [35–37].

As outlined in Table 2, the majority of SNPs accumulated in genes associated with metabolism, most notably in *hem*E and *hem*N genes that are involved in heme metabolism [38]. In prokaryotes, heme is an integral part of proteins involved in multiple electron transport chains for respiration, and a cofactor of many enzymes including catalases, peroxidases, and P(450) class cytochromes [39]. Further analysis including genome sequences of more strains from different hosts and regions will be necessary to elucidate possible functional consequences of the present observation. Similarly, the present dataset cannot provide an explanation why gene GM000264, which is coding for a hypothetical protein of unknown function, was found to accumulate SNPs and have the highest dn/ds ratio (0.526) among all analyzed genes.

In contrast to the high number of SNPs observed in the metabolic genes outlined in Table 2, the T3SS and *pmp* genes were unexpectedly conserved between the two *C. gallinacea* strains with less than 1% sequence dissimilarity. Such congruence and conservation in these genomic regions is in stark contrast to the high genetic variation (up to 15%) in these genes of other chlamydial species [28, 29]. The chlamydial T3SS, a system of structural, chaperone and secreted effector proteins, is considered as “virulence machinery” with a function to deliver effector proteins in order to subvert host cellular processes [40, 41]. As such, genetic variation and polymorphisms in these genes are probably associated with differences in virulence and host and/or tissue tropism, as previously observed for *C. pecorum*, *C. trachomatis*, *C. psittaci* and other chlamydial species [28, 42, 43]. Whether the sequence conservation in T3SS genes is present throughout the *C. gallinacea* taxon remains to be investigated, as well as their role in virulence of this organism. Equally, we further need to investigate the role in infection of the 37 putative *C. gallinacea* Incs identified in this study, and how genetically diverse they will be throughout the taxon.

Highly polymorphic chlamydial *pmp* gene families account for the majority of chromosomal SNPs in other related species. Generally speaking, the *pmp* loci constitute almost 4% of the chlamydial genome, thus suggesting an important biological function due to their adhesive as well as antigenic properties [32]. In this study, the *pmp* genes of *C. gallinacea* somewhat surprisingly displayed sequence conservation, although we observed non-synonymous changes and indels between the two strains. Proteins *Pmp*B, A, E1, H, G1 and G2 appear to be intact in both *C. gallinacea* strains, while strain JX-1 harbored a premature STOP codon in *pmp*D, which will require further investigations to assess whether the protein’s function has been impaired. The remaining *pmp* genes, although truncated, appear to be PmpG paralogs based on our blast and phylogenetic analyses. Based on these (early) observations, our current hypothesis is that *pmp*G expansion may not be necessary for virulence or biology of *C. gallinacea*. Studies on *C. pneumoniae*, where Pmps (including at least 13 *pmp*G subtypes) represent major proteins in the outer membrane, showed that all *pmp* genes are transcribed and expressed during the infectious cycle [32, 44]. In *C. psittaci*, several *pmp*G genes that may be related to host tropism and virulence were identified. It is possible that the *pmp*G group plays a major role in host range, tissue tropism and virulence for different *C. psittaci* strains due to their high diversity and rapid evolution [45, 46]. Considering the importance of *pmps* in chlamydial genomes, the observed non-synonymous changes and indels between the two analyzed strains render future investigations on *pmp* diversity within the species of *C. gallinacea* highly pertinent.

The PZs of the two *C. gallinacea* strains proved compact and highly conserved, with 99.2% sequence similarity, with Mac/Perforin and Phospholipase D genes missing, but harboring the full-length chlamydial cytotoxin (*tox*) gene [47]. *C. gallinacea* JX-1 had a premature STOP codon in the N-terminal regions of the *tox* gene, a region that contain a catalytic glycosyltransferase domain, while *tox* was intact in *C. gallinacea* type strain 08-1274/3. However, whether this has an impact on the function of the *tox* gene remains to be elucidated. Chlamydial *tox* genes are considered important virulence factors and were associated with acute infection and disease [48]. They appear to be a species and niche-specific feature due to their full-length presence in only five related species, including *C. psittaci*, *C. felis*, *C. caviae* with a single gene copy, and *C. pecorum* and *C. muridarum* with two and three copies, respectively [9, 27]. In contrast, *tox* is partially truncated in *C. trachomatis*, while *C. avium*, *C. abortus* and *C. pneumoniae* do not have a *tox* gene. It will be interesting to see how the presence or absence of *tox* gene(s) in chlamydial pathogens can be correlated with virulence properties in the future.

Both *C. gallinacea* strains were found to carry the highly conserved plasmid. In the present study, the plasmid was only detected in 24 *C. gallinacea*-positive samples from chickens out of total 45 samples tested from different avian. Its absence in strains infecting other hosts could be due to a) the plasmid being host specific; and b) more likely, failure to detect it because of low DNA concentration and rapid degradation, considering that chlamydial plasmids are present in low copy numbers (1–10 per chromosome) [49]. Another possibility leading to failure of plasmid detection in clinical samples might be associated with high temperature during DNA extraction. In order to increase the efficiency of DNA extraction from clinical samples, we usually give high temperature (72 °C) and a long time for proteinase K incubation and for the multiple elution steps. This approach increases sensitivity for PCR-based clinical diagnostics, but may induce DNA breakage which probably result in failure of detecting the 7.49 kbp plasmid. The presence of a plasmid is a frequent feature of *Chlamydia* spp. genomes, but naturally occurring plasmid-less strains can also be found [29]. Species like *C. abortus* and (human) *C. pneumoniae* do not carry plasmids at all [50]. Both the role and distribution of the plasmid in *C. gallinacea* strains warrant further investigations.

Previous data from *omp*A genotyping indicated considerable genetic diversity of *C. gallinacea* field strains [4]. The present MLST confirmed this by identifying 15 novel STs among 25 strains.

An analysis of the *C. gallinacea* MLST data allows us to make some speculation on the epidemiology of this pathogen. Identification of the same sequence type in cloacal and oral sites (e.g. A/Ch_40: oral and cloaca, both ST 166) indicates that at least one possible transmission route could be fecal-oral (cloacal). The observations above also indicated transmission among different avian hosts in the same area (e.g. J/Hen 31, 12 and 4 strains, all ST 163) or geographically distinct areas (e.g. JX-1, J/ChA2432, J/ChA2360 and A/Ch29-1 strains, all ST 155). Moreover, there is probably transmission across different hosts, as *C. gallinacea* strains have been frequently isolated from birds and were also found in livestock that have been in contact [2].

While we could not apply MLST on *C. gallinacea* strains from ducks, geese and pigeons, nor to European and livestock strains, we do anticipate that future typing studies will shed more light on the complex epidemiology and genetic diversity of this chlamydial agent.

## Conclusions

Awareness is growing that *C. gallinacea* infections in avian hosts are globally highly prevalent. In the present study, we have provided new evidence that this pathogen is genetically diverse, even though it is still open how these genetic differences among strains infecting chickens and other hosts translate into its biology and pathogenicity. Future studies should include analysis of strains from a variety of avian and other livestock hosts to enable us to understand the evolution and host adaption of this enigmatic pathogen.

## Additional files

Additional file 1: Table S1. *C. gallinacea*-positive samples used for MLST and plasmid detection. (DOCX 13 kb)

Additional file 2: Table S2. Primers used for generating *C. gallinacea* plasmid fragments in this study. (DOCX 14 kb)

Additional file 3: Table S3. Primers used for MLST of *C. gallinacea* in this study. (DOCX 14 kb)

Additional file 4: Table S4. PCR conditions for amplifying seven HK genes in this study. (DOCX 12 kb)

Additional file 5: Table S5. Original data for MLST analysis of the *C. gallinacea*-positive samples. (XLSX 10 kb)

Additional file 6: Table S6. Original data for T3SS of *C. gallinacea*, including the locus-tags, and their annotation/putative function, length and direction. (XLS 36 kb)

Additional file 7: Table S7. List of the predicted transmembrane helices in the analyzed putative *C. gallinacea* inclusion (Inc) proteins from this study. (XLSX 10 kb)

Additional file 8: Figure S1. Graphical representation of the predicted transmembrane helices in the analyzed putative *C. gallinacea* inclusion (Inc) proteins from this study. (DOCX 423 kb)

## Abbreviations

MLST: Multilocus sequence typing
*pmp*: Polymorphic membrane protein
PZ: Plasticity zone
*tox*: *C*ytotoxin
