# Supplementary material for: From genomes to genotypes: molecular epidemiological analysis of Chlamydia gallinacea reveals a high level of genetic diversity for this newly emerging chlamydial pathogen
Source: BMC Genomics. 2017 Dec 6;18:949. doi: 10.1186/s12864-017-4343-9 (PMC5717833; doi:10.1186/s12864-017-4343-9)
Supplement: Supplementary file 1 — C. gallinacea-positive samples used for MLST and plasmid detection. (DOCX 13 kb) [file 12864_2017_4343_MOESM1_ESM.docx]

**Table S1. *C. gallinacea*-positive samples used for MLST and plasmid detection.**

| **Host** | **Province** | **Anatomical site**  **Swab** | **Sample** |
| --- | --- | --- | --- |
| Chicken | Anhui | Oral | **29-1**, **40-1*** |
|  |  | Cloacal | **40-2** |
|  | Guangdong | Cloacal | **A1048**, **A1059**, **A1061**, **A1082** |
|  | Guangxi | Cloacal | **A612** |
|  | Hainan | Cloacal | **A3254**, **A3274** |
|  | Jiangxi | Oral | **A2360** |
|  |  | Cloacal | **A2432** |
|  | Shandong | Oral | **A1403** |
|  |  | Cloacal | **A1469** |
|  | Sichuan | Cloacal | **A325** |
|  | Yunnan | Cloacal | **A3109**, **A3141**, **A3142**, **A3148**, **A3151** |
| Hen | Jiangsu | Cloacal | **4**, **12**, **31**, 34 |
| Duck | Anhui | Oral | 6-1 |
|  |  | Cloacal | 7-2, 8-2 |
|  | Guangdong | Cloacal | A1086, A1088 |
|  | Henan | cloacal | A1361 |
|  | Jiangsu | cloacal | A3626 |
|  | Jiangxi | oral | A3665, A3692, A3694 |
|  | Shandong | oral | A1430 |
|  |  | cloacal | A1498 |
| Pigeon | Hebei | cloacal | A801 |
|  | Jiangsu | oral | A41, A43, A48 |
|  |  | cloacal | A3413, A3415, |
| Goose | Anhui | cloacal | 49-2 |
|  | Jiangxi | oral | A2400, A3518 |

***** Samples in bold denote these MLST-typed samples.
