# Supplementary material for: From genomes to genotypes: molecular epidemiological analysis of Chlamydia gallinacea reveals a high level of genetic diversity for this newly emerging chlamydial pathogen
Source: BMC Genomics. 2017 Dec 6;18:949. doi: 10.1186/s12864-017-4343-9 (PMC5717833; doi:10.1186/s12864-017-4343-9)
Supplement: Supplementary file 2 — Primers used for generating C. gallinacea plasmid fragments in this study. (DOCX 14 kb) [file 12864_2017_4343_MOESM2_ESM.docx]

**Table S2. Primers used for generating *C. gallinacea* plasmid fragments in this study.**

| Regions of the plasmid | Primers | Sequence (5’– 3’) | Amplicon size (bp) |
| --- | --- | --- | --- |
| 4-664bp | plaF1 | TTTTTGACTGTATCGGAAGCTGC | 661 |
|  | plaR1 | TTGCTACTTGTCGACCATTACCC |  |
| 603-1027bp | plaF2 | AGATTATATTGGGAATAGAACTGGCTG | 425 |
|  | plaR2 | ATCATGAAAGTTACAGGGCATTCTT |  |
| 829-1383bp | plaF15 | CTTTCAGCATACGATACTTCCATTGA | 555 |
|  | plaR15 | GCTCAAACTTTATTACAAGGAGCGA |  |
| 1028-1677bp | plaF3 | ATCGGAATCAGAACACCCCATT | 650 |
|  | plaR3 | TTCGCCATGCTGAATCATAACTTA |  |
| 1592-2123bp | plaF4 | TGTTTCGTCCCTTCAGACCATG | 532 |
|  | plaR4 | ATTATCACAGTAATTCACAGCTTGGC |  |
| 2040-2758bp | plaF5 | ATGATAGAGCAATCGGCAGAAGAA | 719 |
|  | plaR5 | ATCTTCAATGCCATGTCGATAGC |  |
| 2641-3488bp | plaF6 | CAGGATACTCATCCATAGATGAACATAGT | 848 |
|  | plaR6 | CTTCTGATAGTAGAGGCTTTCTTCCAA |  |
| 3399-4080bp | plaF7 | gaatcatggtaaaatcagaaaatcagg | 682 |
|  | plaR7 | ATTTCCTGATACATATTAGCTGGTTTGA |  |
| 3995-4672bp | plaF8 | ggattcatcatagaaccatgtccaa | 678 |
|  | plaR8 | ATCAACTTTAATACCATCTGTTGCTTGA |  |
| 4594-4948bp | plaF16 | AGACCAACAAATTATCATCGGAACTT | 355 |
|  | plaR16 | TTCTGTTCCTCCATCAAGAGTATCG |  |
| 4673-5281bp | plaF9 | ATCACCAATAGTAACAATCAAGATGCA | 609 |
|  | plaR9 | TTTGACTTCCAAGAAAGATACGTTTG |  |
| 5190-5851bp | plaF10 | ATAGTGGCGTAGTATGGGTTAATGC | 662 |
|  | plaR10 | TCCACAAGCAAAACTCGCTTCT |  |
| 5775-6446bp | plaF11 | ACTGGAAAAACAACCTTGTCCCTA | 672 |
|  | plaR11 | ATCTTGGGAAGCTCTAGAATTGGG |  |
| 6282-6704bp | plaF17 | GATGAAAGAAACTCAACAAACTCCAC | 423 |
|  | plaR17 | GCGGCTAAATTGTGATTAACGTC |  |
| 6447-7043bp | plaF12 | ATCCTAAAATTGACCGAAGAAATAGAA | 597 |
|  | plaR12 | ATAGCTAAAGTATTAGACATCCCTCGAAT |  |
| 6946-37bp | plaF14 | TACAAAACGGCATATTTATTAGCCTC | 583 |
|  | plaR14 | ACCATACATTAGCAGCTTCCGATAC |  |
