# Supplementary material for: From genomes to genotypes: molecular epidemiological analysis of Chlamydia gallinacea reveals a high level of genetic diversity for this newly emerging chlamydial pathogen
Source: BMC Genomics. 2017 Dec 6;18:949. doi: 10.1186/s12864-017-4343-9 (PMC5717833; doi:10.1186/s12864-017-4343-9)
Supplement: Supplementary file 3 — Primers used for MLST of C. gallinacea in this study. (DOCX 14 kb) [file 12864_2017_4343_MOESM3_ESM.docx]

**Table S3. Primers used for MLST of *C. gallinacea* in this study.**

| Target genes | Locus tag | Primer | Sequence (5’– 3’) | *T*_m_^o^C | Amplicon  size (bp) | MLST fragment (bp) |
| --- | --- | --- | --- | --- | --- | --- |
| ***gat*A**  (Glutamyl-tRNA amidotransferase) | GM000463 | gatF | GCCTTAGAGTTAAGAAATGCTGTGG | 58 | 513 | 425 |
|  |  | gatR | CGATCCCCCAGTGTCTGAGC |  |  |  |
| ***hfl*X**  (GTP-binding protein) | GM000478 | hflF | GAAGAAATTACAGCTTCACAGCAGAG | 60 | 643 | 438 |
|  |  | hflR | CCAAAGGATGGGAAGCATCG |  |  |  |
| ***gid*A**  (Glucose-inhibited division protein A) | GM000628 | gidF | GGAGTCACAACTAAAGAAGGCATTG | 62 | 559 | 476 |
|  |  | gidR | GGTATTGAACATCAAAGGGCATTG |  |  |  |
| ***eno*A**  (Enolase) | GM000800 | enoF | TCATGATAGAAACCGATGGCACT | 58 | 695 | 381 |
|  |  | enoR | CTTTATGAATCCCTTCGGCAATG |  |  |  |
| ***opp*A_3**  (Oligobinding protein) | GM000228 | oppF | AGATACCATTGGGAGTCAGTTTTG | 58 | 606 | 483 |
|  |  | oppR | TAGGGTAAAATGCACCGTTCG |  |  |  |
| ***hem*N**  (Coproporphyrinogen III oxidase) | GM000887 | hemF | ATTGCTATTGAATTTGATCCACGT | 58 | 572 | 432 |
|  |  | hemR | CTTGTCGCTGTAATTCCTAATCCTA |  |  |  |
| ***fum*C**  (Fumarate hydratase class II) | GM000055 | fumF | GCCTCGTGAAGTGATTCAAGCT | 62 | 652 | 465 |
|  |  | fumR | AGGCTCACCTGTTTCCTTACGT |  |  |  |
