# Supplementary material for: From genomes to genotypes: molecular epidemiological analysis of Chlamydia gallinacea reveals a high level of genetic diversity for this newly emerging chlamydial pathogen
Source: BMC Genomics. 2017 Dec 6;18:949. doi: 10.1186/s12864-017-4343-9 (PMC5717833; doi:10.1186/s12864-017-4343-9)
Supplement: Supplementary file 4 — PCR conditions for amplifying seven HK genes in this study. (DOCX 12 kb) [file 12864_2017_4343_MOESM4_ESM.docx]

Table S4. PCR conditions for amplifying seven HK genes in this study.

| Thermal protocols | 6 cycles, 1 sec @ 95°C, 12 sec @64°C, 8 sec @ 72°C |
| --- | --- |
|  | 9 cycles, 1 sec @ 95°C, 12 sec @ 62°C, 8 sec @ 72°C |
|  | 3 cycles, 1 sec @ 95°C, 12 sec @ 60°C, 8 sec @ 72°C |
|  | 30 cycles, 30 x 1 sec @ 95°C, 12 sec @ 54°C, 30 sec @ 67°C, and 10 sec @ 72°C |
| PCR components for a single PCR of 20 μl | 10.0 μl DNA template, 0.2μl forward primer (100 μM), 0.2μl reverse primer (100 μM), 4.0 μl 5 × PCR buffer, 0.4 μl 10 μM dNTP, 0.3 μl 5 U/μl *Taq* DNA polymerase and 4.9 μl ultrapure H_2_O. |
