# Supplementary material for: From genomes to genotypes: molecular epidemiological analysis of Chlamydia gallinacea reveals a high level of genetic diversity for this newly emerging chlamydial pathogen
Source: BMC Genomics. 2017 Dec 6;18:949. doi: 10.1186/s12864-017-4343-9 (PMC5717833; doi:10.1186/s12864-017-4343-9)
Supplement: Supplementary file 8 — Graphical representation of the predicted transmembrane helices in the analyzed putative C. gallinacea inclusion (Inc) proteins from this study. (DOCX 423 kb) [file 12864_2017_4343_MOESM8_ESM.docx]

**
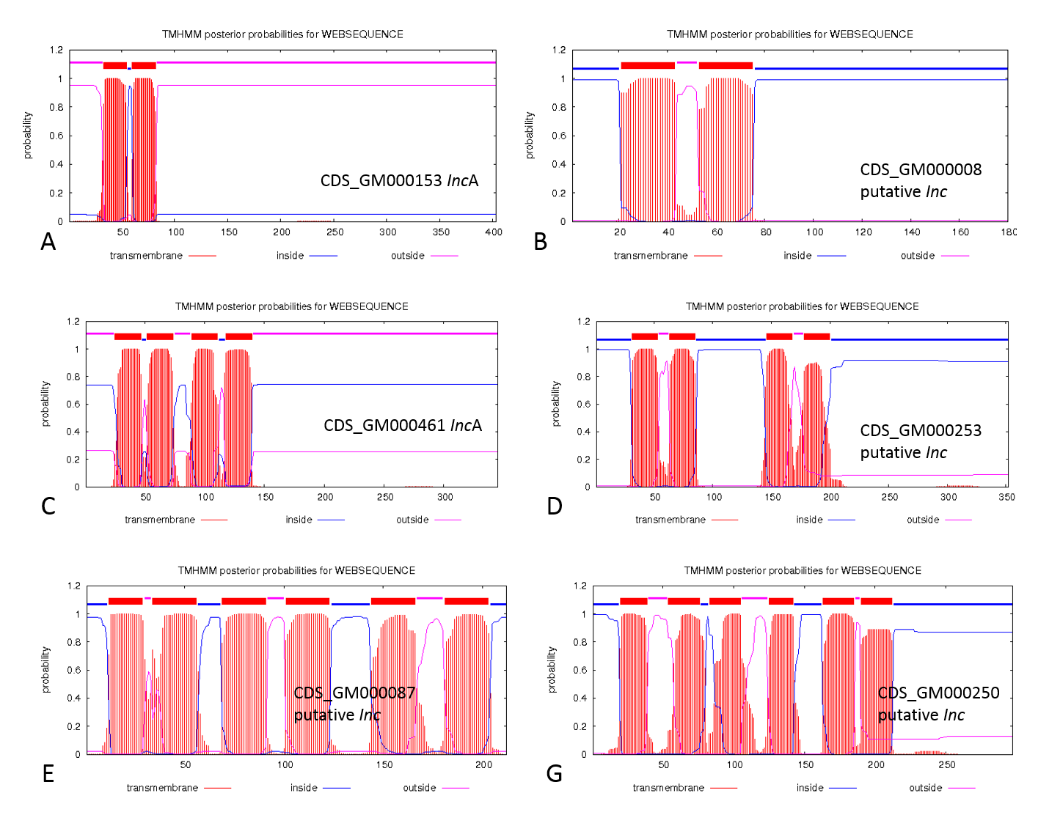
**

**Figure S1. Graphical representation of the predicted transmembrane helices in the analyzed putative *C. gallinacea* inclusion (Inc) proteins from this study.** Structure of the A) *C. gallinacea* CDS_GM000153, and B) *C. gallinacea* CDS_GM000008 characterized with a single bilobed transmembrane (TM) domain; C) *C. gallinacea* CDS_GM000461, and D) *C. gallinacea* CDS_GM000253 characterized with two bilobed transmembrane (TM) domains; E) *C. gallinacea* CDS_GM000087, and F) *C. gallinacea* CDS_GM000250 characterized with three bilobed transmembrane (TM) domains. On the X axis is the number of amino acid at the particular position, while on the Y axis is the probability for the domain.
